# Proteinuria is common among HIV patients: what are we missing?

**DOI:** 10.6061/clinics/2015(10)06

**Published:** 2015-10

**Authors:** Vicente Sperb Antonello, Ivan Carlos Ferreira Antonello, Sandra Herrmann, Cristiane Valle Tovo

**Affiliations:** I Hospital Fêmina, Controle e Prevenção do Departamento de Infecção, Porto Alegre, RS, Brazil; II Universidade Federal de Ciências da Saúde de Porto Alegre, Curso de Pós Graduação em Hepatologia, Porto Alegre, RS, Brazil; III Pontifícia Universidade Católica do Rio Grande do Sul, Programa de Graduação de Medicina e Ciências da Saúde, Porto Alegre, RS, Brazil; IV Mayo Clinic College of Medicine, Division of Nephrology and Hypertension, Rochester, MN, USA

**Keywords:** Proteinuria, Chronic Kidney Disease, HIV, Hepatitis C Virus, Risk Factors, Epidemiology

## Abstract

**OBJECTIVES:**

HIV-related renal diseases are the leading causes of chronic kidney diseases worldwide. The present study aimed to investigate the prevalence of pathological proteinuria and its risk factors among HIV patients.

**METHODS:**

A review of the medical records of 666 HIV-infected individuals aged 18 years or older in an urban HIV/AIDS clinic based in Porto Alegre in southern Brazil. Overt proteinuria was defined as a protein-to-creatinine ratio greater than 150 mg/g according to Kidney Disease: Improving Global Outcomes.

**RESULTS:**

The prevalence of pathological proteinuria in the present study cohort was 20%. Characteristics associated with pathological proteinuria after univariate analysis included alcohol abuse, hepatitis C virus coinfection, the occurrence of diabetes and therapy including tenofovir. Adjusted residuals analysis indicated an association between pathological proteinuria and both a CD4 lymphocyte count below 200 cells/mm^3^ and a viral load higher than 1000 copies/mL. Additionally, an absence of pathological proteinuria was associated with a CD4 lymphocyte count higher than 500 cells/mm^3^. After adjustment for variables with *p*<0.2 in the univariate analysis using a Poisson regression model, tenofovir-containing regimens and a CD4 lymphocyte count below 200 cells/mm^3^ were significantly associated with pathological proteinuria.

**CONCLUSION:**

The risk of chronic kidney diseases in this large contemporary cohort of HIV-infected individuals appeared to be attributable to a combination of HIV-related risk factors. In addition to the traditional risk factors cited in the literature, both regimens containing tenofovir and HIV disease severity seem to be associated with chronic kidney diseases in patients with HIV. Assessment of proteinuria constitutes a novel method for chronic kidney disease staging in HIV-infected individuals and may be effectively used to stratify the risk of progression to end-stage renal disease.

## INTRODUCTION

HIV-related renal diseases are one of the leading causes of chronic kidney diseases (CKDs) worldwide. In particular, the new era of highly active antiretroviral therapy (HAART) and early HIV diagnosis has improved survival and disease progression, leading to a higher proportion of patients with renal abnormalities over time 1-3.

CKD is defined by a sustained change in urinary sediment, such as the presence of proteinuria, or by a reduced glomerular filtration rate (GFR). Proteinuria is often the earliest manifestation of CKD and is more common in HIV-infected individuals than in similarly aged HIV-negative controls 1,4,5. Risk factors for incident or progressive CKD in HIV-positive adults include apolipoprotein-1 genetic polymorphism 4, hepatitis C virus (HCV) coinfection 5, a low CD4 T-cell count, a high HIV viral load and traditional CKD risk factors such as diabetes and hypertension 6.

The present study aimed to investigate the prevalence of pathological proteinuria and its risk factors among HIV patients from a public HIV/AIDS reference clinic in Porto Alegre, Brazil.

## MATERIALS AND METHODS

The present research was a cross-sectional study of HIV-infected patients who attended a public HIV/AIDS clinic in Porto Alegre, Brazil. The data were collected from the records of patients who attended between March 2008 and December 2012. The study population included HIV patients aged 18 years or older who were from the city of Porto Alegre and surrounding metropolitan region. All participants had free access to health care and medications, in line with the national health program. Cases were excluded if records were not available for analysis.

Data collected from records included age, gender, ethnicity, body mass index (BMI), smoking history, use of illicit drugs and alcohol, previous diagnosis of hypertension, diabetes mellitus, chronic hepatitis B infection, chronic HCV infection, lipid levels, glucose levels, urinalysis results, arterial hypertension, drugs being used, current CD4 count (cells/mm^3^), current plasma HIV RNA level (viral load), absence (*naïve*) or use of HAART and type of HAART under use.

Urine samples were analyzed by the Department of Laboratory Medicine at the Universidade Federal do Rio Grande do Sul, Porto Alegre, Brazil and the diagnosis of proteinuria was made based on the urinary protein-to-creatinine ratio (PCR) in a single-spot urine analysis 3. Overt proteinuria was defined as a PCR greater than 150 mg/g according to Kidney Disease: Improving Global Outcomes (KDIGO) 7.

Blood pressure was determined based on an average of two measurements using a calibrated automated machine, with each participant sitting in a relaxed, upright position. Hypertension was defined according to standard definitions set out in the Eighth Joint National Committee guidelines 8. Patients receiving an antihypertensive medication, irrespective of blood pressure, were also defined as hypertensive 8. The criteria for the definitions of diabetes and dyslipidemia followed ADA 9 and AACE 10 guidelines.

Statistical analyses included descriptive statistics with numbers (proportions) for categorical variables and means (± standard deviation) for continuous variables. Categorical variables were assessed using the chi-square test or Fisher's exact test and quantitative variables were compared using Student's t-test. Adjusted residual analysis was performed to detect categories with a two-tailed (higher or lower) expected frequency.

Significant variables with *p*<0.20 in the univariate analysis were further analyzed using a Poisson regression model with robust variance. Fosamprenavir plus ritonavir (FPV/r) treatment was not included in this model due to its use by only a small number of patients. A value of *p*<0.05 was considered statistically significant. All statistical analyses were performed using the SPSS software, version 18 (IBM, Armonk, NY, USA).

### Ethics

The present research was approved by the Ethics Committee of the Municipal Council of Health of Porto Alegre city, under protocol number 05773912.1.0000.5338, in January 2013.

## RESULTS

### Demographics

Initially, 744 HIV-infected individuals were identified, but 78 were excluded due to a lack of complete medical records. Therefore, the medical records of 666 HIV-infected individuals aged 18 years or older in an urban HIV/AIDS clinic based in Porto Alegre in southern Brazil were ultimately evaluated. The mean age of the group was 42.02±11.8 years and 51.5% were male. Moreover, 60.7% of the individuals were Caucasian and 39.3% were Black. Diabetes mellitus was present in only 5.9% of the study population, whereas dyslipidemia was present in 26.2%. A smoking habit was present in 39.3%, and 13.7% of evaluated patients had a BMI higher than 30 kg/m^2^. In terms of the CD4 count, 10.5% had a current count <200 cells/mm^3^; 42.4%, between 200 and 500 cells/mm^3^; and 47.1%, >500 cells/mm^3^. In total, 60% had an undetectable viral load (<50 copies/mL) and 77.7% were currently receiving HAART. Finally, only two patients had serum creatinine greater than 1.5 mg/dL. The group demographic values are presented in Table 1. The prevalence of hypertension in this study cohort was 22.5% (227 individuals). A total of 150 patients were taking antihypertensive drugs. More specifically, 77 individuals (51.33%) were taking only one antihypertensive, 50 (33.33%) were taking two and 23 (15.33%) were taking a combination of three or more drugs. The most common antihypertensive medications were angiotensin-converting enzyme inhibitors (n=100), diuretics (n=61), beta-blockers (n=47), angiotensin II receptor antagonists (n=20) and calcium channel blockers (n=12). In contrast, in the studied group, 61 (40.66%) individuals did not have controlled blood pressure, despite antihypertensive therapy.

### Characteristics associated with pathological proteinuria

The prevalence of pathological proteinuria in the present study cohort was 20% (95% CI: 16.9% to 23.0%), with no difference observed between the groups receiving HAART (19.2%) and not receiving HAART (20.7%) (*p*=0.773). The mean ages of the individuals in the pathological proteinuria group and the non-pathological proteinuria group were 45.8±12.7 years and 41.1±11.4 years, respectively (*p*=0.003). Characteristics associated with pathological proteinuria after univariate analysis included alcohol abuse (*p=*0.019), HCV coinfection (*p*<0.001) and the occurrence of diabetes (*p*=0.006). Therapy including tenofovir among individuals under HAART presented a similar association (*p*<0.001). Adjusted residuals analysis indicated an association between pathological proteinuria and both a CD4 lymphocyte count below 200 cells/mm^3^ (*p*<0.001) and viral load higher than 1000 copies/mL (*p*=0.026). Additionally, an absence of pathological proteinuria was associated with a CD4 lymphocyte count higher than 500 cells/mm^3^ (*p*<0.001).

When the individuals under tenofovir therapy were evaluated regarding proteinuria, 30.11% (53/176) patients had pathological proteinuria and only 20.75% (11/53) of these individuals had albuminuria detected by dipstick.

After adjustment for all variables with *p*<0.2 in the univariate analysis using a Poisson regression model, tenofovir-containing regimens and a CD4 lymphocyte count below 200 cells/mm^3^ were significantly associated with pathological proteinuria. Additionally, within this setting, a viral load higher than 1,000 copies/mL (*p*=0.065) showed a tendency toward association with pathological proteinuria. The regression model values are shown in Table 2.

## DISCUSSION

Proteinuria is part of the definition of CKD and is a risk marker for progression to end-stage renal disease (ESRD). HIV-associated nephropathy (HIVAN)-related risk factors, including an elevated HIV RNA level, a low absolute CD4 lymphocyte count, apolipoprotein-1 genetic polymorphism and hepatitis C and hepatitis B coinfection, are associated with proteinuria 2. The present study detected a high prevalence of pathological proteinuria (20%) among HIV-infected individuals, similar to other studies in the literature, which have reported prevalence values ranging from 17% to 32% 1,11.

This study examined the factors associated with proteinuria within a large cohort of HIV-infected patients. Proteinuria was specifically associated with an elevated HIV RNA level and a CD4 lymphocyte count below 200 cells/mm^3^. These findings are consistent with prior studies, in which risk factors for incident or progressive CKD in HIV-positive adults included HIV disease severity 1,7,. However, factors traditionally associated with CKD that have been described in the literature, such as age, cardiovascular disease 1,4, diabetes, hypertension, obesity 6,12 and HCV 13, were not linked to pathological proteinuria among HIV-positive individuals in the current study after multivariate analysis.

The relationship between antiretrovirals and pathological proteinuria was also examined and tenofovir-based therapy was significantly associated with this condition in the multivariate analysis. Most of the individuals in this group who presented with pathological proteinuria did not have albuminuria detected by dipstick (79.25%), suggesting proteinuria of tubular origin. Thus, these patients would not have benefited from using angiotensin-converting enzyme inhibitors or angiotensin receptor blockers. Meanwhile, FPV/r therapy showed a tendency toward association with pathological proteinuria. However, due to the small number of patients, fosamprenavir was not included in the multivariate analysis. All other antiretrovirals were not linked to pathological proteinuria.

Patients with HIV infection are at risk of developing nephrotoxicity in response to antiretrovirals. In fact, tenofovir and boosted protease inhibitors have been associated with a decline in the GFR 14,15. Tenofovir is specifically a nucleoside reverse transcriptase inhibitor that can cause acute kidney injury (AKI), proximal tubular dysfunction, or both in combination. The risk of kidney toxicity with tenofovir has varied across different studies, with estimates ranging from 2% to 10% 15,16.

The benefits of antiretrovirals concerning proteinuria may be less substantial with regimens containing tenofovir compared with the alternative, namely, nucleoside reverse transcriptase inhibitors 17. Although a kidney biopsy may not be necessary to diagnose tenofovir toxicity, it can be useful in cases where the diagnosis is less clear or where there are compelling reasons not to discontinue tenofovir 18. In addition, in the setting of renal disease, a regimen containing a boosted protease inhibitor (such as FPV/r) in combination with tenofovir has been associated with a decrease in the GFR 14, 19.

The present study has certain limitations. For example, several of our findings could have been underestimated, given that the durations of HIV disease and HAART were not evaluated. Additionally, our analyses were based on a cross-sectional study; hence, the temporality between factors and the development of pathological proteinuria could not be established. It was also difficult to estimate the number of patients with CKD due to the small proportion of patients with abnormal serum creatinine.

Conversely, our study possessed certain strengths as well. In particular, this study was conducted in a population of HIV-infected persons with clinical follow-up and reliable information concerning antiretroviral use. The group studied was representative for two reasons: a large cohort of patients was evaluated and the profile of the patients within the group is similar to that of patients currently attending HIV clinics.

In conclusion, the risk of CKD in this large contemporary cohort of HIV-infected individuals appeared to be attributable to a combination of HIV-related risk factors. In addition to the traditional risk factors cited in the literature, both regimens containing tenofovir and HIV disease severity seem to be very important factors associated with CKD in patients with HIV.

Assessment of proteinuria constitutes a novel method for CKD staging in HIV-infected individuals and may be effectively used to stratify the risk of progression to ESRD. Given the increasing incidence of HIV-related renal diseases as a cause of ESRD and the increased mortality risk that ESRD imparts, further investigation of strategies to prevent or treat the renal complications of HIV infection is imperative.

## Figures and Tables

**Table 1:** Demographic data comparing groups of pathological proteinuria and non-pathological proteinuria individuals.

Factors	N	Total (%)	Pathological Proteinuria Group	Non-Pathological Proteinuria Group	*p* value
**Population size**		666 (100%)	133 (20.0)	533 (80.0)	-
**Age, years**	666				0.003
18-39		288 (43.2)	42 (31.6)	246 (46.2)	
> 40		378 (56.0)	91 (68.4)	287 (53.8)	
**Gender, male**	666	343 (51.5)	62 (46.6)	281 (52.7)	0.245
**Ethnicity**	603				
Caucasian		366 (60.7)	71 (57.7)	295 (61.5)	0.514
**Abuse on drugs**					
Alcohol	635	92 (14.5)	26 (21.7)	66 (12.8)	0.019
Tabaco	634	249 (39.3)	54 (45.5)	195 (37.9)	0.159
Crack cocaine	635	24 (3.8)	6 (5.0)	18 (3.5)	0.431
Inhaled cocaine	634	21 (3.3)	2 (1.7)	19 (3.7)	0.396
Cannabis	634	19 (3.0)	4 (3.3)	15 (2.9)	0.769
**Body mass index**	408				
Obese (≥ 30.0)		56 (13.7)	12 (15.2)	44 (13.4)	0.811
**Diabetes mellitus**	665	39 (5.9)	15 (11.3)	24 (4.5)	0.006
**Dyslipidemia**	665	174 (26.2)	35 (26.3)	139 (26.1)	1
**Hypertension**	659	142 (21.5)	36 (27.1)	106 (20.2)	0.106
**Chronic hepatitis B**	666	20 (3.0)	7 (5.3)	13 (2.4)	0.094
**Chronic hepatitis C**	664	80 (12.0)	29 (21.8)	51 (9.6)	< 0.001
**Current CD4 count, cells/mm3**	665				0.001
≥500		313 (47.1)	47 (35.3)	266 (50.0)	
200-500		282 (42.4)	62 (46.6)	220 (41.4)	
< 200		70 (10.5)	24 (18.0)	46 (8.6)	
**HIV RNA, copies/mL**	666				0.026
< 50		398 (59.8)	76 (57.1)	322 (60.4)	
50-1,000		110 (16.4)	15 (11.3)	95 (17.8)	
>1,000		158 (23.7)	42 (31.6)	116 (21.8)	
**HAART regimen**	651				
Yes		506 (77.7)	97 (76.4)	409 (78.1)	0.773
**Current use of NRTI or NOT**	637				< 0.001
TDF+3TC		175 (27.5)	52 (43.3)	123 (23.8)	
AZT+3TC		317 (49.8)	38 (31.7)	279 (54)	
NAIVE		145 (22.8)	30 (25)	115 (22.2)	
**Based-therapy**	666				
**NNRTI**					
Efavirenz		197 (29.6)	37 (27.8)	160 (30.0)	0.696
Nevirapine		6 (0.9)	1 (0.8)	5 (0.9)	1
**Protease inhibitors**					
Lopinavir		155 (23.3)	30 (22.6)	125 (23.5)	0.917
Atazanavir		126 (18.9)	27 (20.3)	99 (18.6)	0.741
Fosamprenavir		14 (2.1)	0 (0.0)	14 (2.6)	0.085
Darunavir		8 (1.2)	2 (1.5)	6 (1.1)	0.663

3TC: Lamivudine; AZT: Zidovudine; HAART: highly active antiretroviral therapy; NRTI: nucleoside reverse transcriptase inhibitors; NNRTI: Non- nucleoside reverse transcriptase inhibitors; TDF: Tenofovir.

**Table 2:** Pathological proteinuria: Poisson regression model of all variables with *p*<0.2 in the univariate analysis.

			95%?Confidence Interval for PR
Variable	*p*-value	Prevalence Rate	Lower	Upper
Age >40 years	0.323	1.242	0.808	1.909
Alcohol abuse	0.342	1.271	0.775	2.083
Smoke	0.433	1.178	0.782	1.774
Diabetes	0.115	1.756	0.873	3.494
Hypertension	0.244	1.323	0.826	2.122
Chronic Hepatitis B	0.264	1.631	0.691	3.850
Chronic Hepatitis C	0.117	1.488	0.905	2.445
Viral load >1,000 copies/mL	0.059	1.543	0.973	2.445
Viral load 51-1,000 copies/mL	0.664	0.878	0.487	1.583
Viral load <50 copies/mL		Ref		
CD4 <200 cells/mm^3^	0.031	1.947	1.062	3.569
CD4 500-200 cells/mm^3^	0.062	1.503	0.980	2.305
CD4 >500 cells/mm^3^		Ref		
Regimen containing tenofovir	0.004	1.880	1.224	2.887
